# A Rare Case Report of Nosocomial Urosepsis due to *Klebsiella pneumoniae* in a Preterm Neonate Complicated by Acute Cystitis

**DOI:** 10.1155/crdi/3455145

**Published:** 2026-09-12

**Authors:** Bormey Sim, Sakada Kok, Sethikar Im, Sakviseth Bin

**Affiliations:** ^1^ Neonatal Intensive Care Unit, Calmette Hospital, Phnom Penh, Cambodia; ^2^ Pediatric Department, University of Health Sciences, Phnom Penh, Cambodia, sbu.edu.tr; ^3^ Office of Secretary of State, Ministry of Health, Phnom Penh, Cambodia, moh.koshi.gov.np

**Keywords:** cystitis, hospital-acquired infection, *Klebsiella pneumoniae*, preterm infants, urosepsis

## Abstract

Late‐onset sepsis secondary to urinary tract infection in preterm infants is frequently underdiagnosed and rarely reported. Establishing this diagnosis remains challenging, as clinical manifestations are often nonspecific, inflammatory markers may be inconclusive, and urinalysis is not routinely performed in many NICUs. We report a case of urosepsis originating from *Klebsiella pneumoniae–*associated cystitis in a 41‐day‐old preterm infant who had been admitted to our NICU since birth. The infant had multiple risk factors for sepsis, including prolonged mechanical ventilation and the presence of an indwelling central line. This case highlights the importance of maintaining a high suspicion index for urinary tract infection in preterm neonates with unexplained clinical deterioration and emphasizes the need for early urine evaluation as part of the sepsis workup.

## 1. Introduction

Late‐onset sepsis (LOS), defined as sepsis occurring after 72 h of life [1], remains a significant cause of morbidity and mortality among neonates, particularly in premature infants with prolonged hospitalization in neonatal intensive care units (NICUs). The incidence of LOS varies widely, ranging from 0.6% to 14%, and is strongly associated with invasive procedures such as central venous catheterization and mechanical ventilation [2, 3]. Among the infectious causes of LOS, urinary tract infections (UTIs) are increasingly recognized, with reported incidence rates between 0.1% and 5.5% in neonates, and a higher susceptibility observed in preterm infants [4, 5]. *Klebsiella* species, especially *Klebsiella pneumoniae*, are among the leading pathogens responsible for nosocomial UTIs and LOS in preterm infants, particularly in low‐resource settings [5–7]. Risk factors such as prolonged NICU stay, exposure to invasive devices, and prior antibiotic therapy contribute to increased vulnerability in this population [8]. Diagnosing neonatal UTI remains challenging due to nonspecific clinical presentations and frequently blunted inflammatory markers [9, 10]. We report a rare case of *Klebsiella pneumoniae* UTI causing LOS in a premature neonate following extended NICU care, highlighting the importance of timely diagnosis and appropriate antimicrobial management in this high‐risk group.

## 2. Case Presentation

A male neonate was born prematurely at 29 weeks and 4 days of gestational age via vaginal delivery, with a birth weight of 1100 g (32nd percentile), a length of 38 cm (45th percentile), and a head circumference of 25 cm (7th percentile). The Apgar scores at 1, 5, and 10 min were 8, 9, and 9, respectively. The mother’s prenatal course was generally unremarkable, with the exception of preterm prelabor rupture of membranes (PPROMs) 6 days prior to delivery and elevated inflammatory markers. These markers included a C‐reactive protein (CRP) level of 69.7 mg/L and significant leukocyturia (3,200,000 cells/mL). The maternal procalcitonin (PCT) level was 0.5 ng/mL, and blood cultures were pending at delivery.

The neonate was admitted to the NICU for respiratory distress syndrome (RDS) and suspected early‐onset sepsis (EOS). Initial sepsis workup, including blood cultures, was negative. The infant required intubation and mechanical ventilation for 7 days and received two doses of surfactant. An umbilical venous catheter (UVC) was placed for 6 days, followed by insertion of a peripherally inserted central catheter (PICC) to facilitate intravenous therapy and parenteral nutrition. The PICC was maintained for 28 days. Empirical first‐line antibiotics were administered for 3 days. In addition, nutritional supplementation and specialized developmental care were provided to support growth and neurodevelopment, acknowledging the critical importance of comprehensive care in preterm infants.

On Day 41 of life (corrected age of 31 weeks and 1 day), within the hospitalization period, the neonate developed fever and feeding intolerance, clinical signs that raised suspicion for a secondary infection. Laboratory investigations revealed leukocytosis (leukocyte count of 20 × 10^9^) and mild thrombocytopenia (platelet count of 121 × 10^9^), as shown in Table 1. Notably, inflammatory markers such as CRP (7.3 mg/L) and PCT (0.28 ng/mL) were within normal limits, illustrating the diagnostic challenge of infection in preterm neonates, where typical markers may be blunted or nonspecific. Blood culture was retaken despite the negativity of inflammatory markers. With a history of prolonged hospitalization, urinalysis was performed as well to rule out possible sources of infection. According to the local sepsis guideline, meropenem and amikacin were initiated. Urine examination revealed significant leukocyturia (26,000 cells/mL), with negative protein and nitrite results. A urine culture performed on the same day isolated *Klebsiella pneumoniae* at 10^5^ colony‐forming units per milliliter. Kidney, ureter, and bladder (KUB) ultrasound demonstrated bladder wall thickening measuring greater than 5 mm, consistent with cystitis, thereby confirming acute cystitis complicated by nosocomial urosepsis.

**TABLE 1:** Laboratory investigations of the neonate.

	At birth	Day 3 of life	Day 10	Day 41 (onset of fever)	Day 44	Day 47
White blood cell (4–9 × 10^9^/L)	30.2	28.4	22.4	20	15.6	18.5
Hemoglobin (13–170 g/L)	16.3	17.9	12.2	20	14.5	14.9
Platelets (150–450 × 10^9^/L)	338	478	579	121	115	370
C‐Reactive protein (< 5 mg/L)		9.67	3.6	7.3	5.1	4.64
Procalcitonin (< 0.5 mcg/L)	0.38		0.24	0.28	0.19	0.126
Blood urea nitrogen (BUN; 4–13 mmol/L)			4	0.23		
Creatinine (44–106 µmol/L)			49	69		
Aspartate aminotransferase (< 31 U/L)			88			
Alanine transaminase (< 34 U/L)			50			
Hemoculture	Neg			Neg	Neg	

Targeted antimicrobial therapy was promptly initiated following the identification of *Klebsiella pneumoniae* and its antibiotic susceptibility profile. The neonate received a 10‐day course of meropenem in combination with 7 days of amikacin. This combination therapy was selected to maximize bactericidal activity while minimizing the risk of resistance development. Serial urine cultures obtained on Day 7 of treatment were sterile, confirming effective microbiological clearance (see Table 2). Clinically, the neonate demonstrated progressive improvement, with resolution of fever and feeding intolerance. He was subsequently discharged 10 days after diagnosis, with a structured outpatient follow‐up plan for ongoing growth and neurodevelopmental monitoring, as well as surveillance for potential recurrence, renal complications, or antimicrobial‐related adverse effects.

**TABLE 2:** Urine examination and cultures.

Urinalysis	Day 41 of life (onset of fever)	Day 47 (antibiotics Day 7)
Physical examination		
Color	Pale yellow	Pale yellow
Appearance	Clear	Clear
Specific gravity (SG)	1.005	1.015
pH	6.5	6.5
Chemical examination		
Protein	Neg	Neg
Glucose	Neg	Neg
Ketone	Neg	Neg
Bilirubin	Neg	Neg
Urobilinogen	Neg	Neg
Nitrite	Neg	Neg
Urine sedimentation		
Leukocytes	26,000/mL	10,000/mL
Red blood cells	00/mL	00/mL
Squamous cells	Rare	Rare

**Uroculture**	***Klebsiella* *pneumoniae***	**Neg**

	100.000 CFU/mL	

## 3. Discussion

Neonatal sepsis remains a major global health problem, with an estimated 1.3 million cases annually and a disproportionately high burden in preterm and low birth weight infants [11]. This burden is especially high in low‐ and middle‐income countries, where limited resources, more premature births, and weaker infection‐prevention systems lead to higher incidence and worse outcomes. Moreover, Gram‐negative organisms, especially *Klebsiella pneumoniae*, now account for a substantial proportion of late‐onset and healthcare‐associated neonatal sepsis episodes worldwide, with several reports from high‐burden regions describing *Klebsiella* spp. as leading pathogens in NICU‐acquired infections [11–13]. In this context, our case of a very preterm neonate who developed nosocomial urosepsis underscores how fragile preterm infants are, who are at particular risk of severe Gram‐negative infections, and highlights the importance of systematically identifying and mitigating modifiable risk factors in low‐income settings.

Regarding risk factors for LOS, preterm infants in the NICU are frequently exposed to multiple invasive procedures that facilitate both colonization and subsequent infection by *Klebsiella pneumoniae* and other multidrug‐resistant (MDR) Gram‐negative organisms [12, 14]. Gut acquisition of extended‐spectrum β‐lactamase (ESBL) producing *Klebsiella* in preterm neonates has been strongly linked to enteral feeding practices, endotracheal intubation, and prolonged use of central vascular catheters (UVC and PICC), emphasizing the central role of NICU care processes in the chain of transmission [15]. A large systematic review analyzing NICU outbreaks over 2 decades reported that bacteria were responsible for the majority of outbreaks (80.3%), with *Klebsiella pneumoniae* being the most commonly identified pathogen. Prematurity and low birth weight were the principal risk factors, while healthcare workers’ hands were identified as the predominant route of transmission [16]. These findings are consistent with our case, in which the patient had several recognized risk factors, including extreme prematurity, RDS requiring a week of mechanical ventilation, and a PICC maintained for nearly 4 weeks, all of which are known to increase the risk of intestinal colonization and late‐onset nosocomial infection with *Klebsiella* spp. Furthermore, maternal premature prelabor rupture of membranes and elevated antenatal inflammatory markers in the case likely contributed to the increased susceptibility to infection, despite the initial EOS evaluation being negative. This illustrates how late‐onset episodes may develop during prolonged hospitalization rather than directly continuing early‐onset disease.

Diagnosing LOS in neonates is challenging worldwide and particularly difficult in resource‐restricted countries, where limited access to advanced laboratory testing, delayed culture results, and overlapping clinical presentations often hinder early recognition [17, 18]. Inflammatory markers such as CRP and PCT, while useful in older children and adults, may be normal or only mildly elevated in preterm infants with invasive infection because their immune response is frequently blunted and nonspecific [12, 19]. Consequently, the case highlights the difficulty in diagnosing LOS in preterm neonates. The infant presented with fever and feeding intolerance, but inflammatory markers (both CRP and PCT) were within the normal range. Significant leukocyturia and isolation of a single microorganism, *Klebsiella pneumoniae*, at a high titer in the urine culture confirmed the presence of infection. This finding underscores the importance of urine testing in preterm infants with prolonged device exposure or other risk factors for infection, since relying solely on blood inflammatory markers may delay diagnosis and treatment. Furthermore, bladder wall thickening greater than 5 mm on ultrasound in the context of pyuria and a positive urine culture is consistent with acute cystitis, although such imaging‐confirmed lower urinary tract involvement is seldom documented in neonatal *Klebsiella* infections, which are more commonly reported as isolated bacteremia or meningitis [12, 13]. Early imaging in this case confirmed cystitis and ruled out upper tract involvement or structural abnormalities, factors that may contribute to recurrent infections and long‐term renal complications in preterm infants.

The management of nosocomial *Klebsiella* infections in the NICU is increasingly complicated by the high prevalence of MDR and ESBL‐producing strains, which limit the effectiveness of standard empiric regimens and often necessitate the use of broader‐spectrum agents [20]. In this case, targeted therapy with a 10‐day course of meropenem combined with 7 days of amikacin, chosen after confirmation of susceptibility on culture, resulted in microbiological clearance on follow‐up urine culture and complete clinical recovery, consistent with reports that substantially improve outcomes even in very preterm neonates with severe nosocomial *Klebsiella* infections [12, 19]. Limitations of this report include that it represents a single case from our NICU, with limited available resources; therefore, generalization of the findings is not possible, and causal inference is limited.

## 4. Conclusion

In conclusion, this case highlights the underrecognized risk of *Klebsiella pneumoniae* urosepsis in extremely preterm infants with prolonged NICU stays and invasive devices. Nonspecific clinical signs and normal inflammatory markers can complicate timely diagnosis, emphasizing the critical role of urine testing and imaging in suspected LOS. Targeted antimicrobial therapy based on susceptibility testing led to successful clinical and microbiological resolution. This report also underscores the importance of stringent infection prevention, careful device management, and antimicrobial stewardship, especially in resource‐limited settings where the burden of LOS remains high.

## Funding

The authors received no financial support for the research, authorship, and/or publication of this article.

## Disclosure

This manuscript is based on work previously presented as a poster at the 43rd Annual Meeting of the European Society for Pediatric Infectious Diseases (ESPID 2025).

## Ethics Statement

No ethical approval is required.

## Consent

Written informed consent was obtained from the patient’s parents and is available for review upon request.

## Conflicts of Interest

The authors declare no conflicts of interest.

## Supporting Information

Additional supporting information can be found online in the Supporting Information section.

## Supporting information


**Supporting Information** CARE checklist.

## Data Availability

The data used to support the findings of this study are available from the corresponding author upon request.
