# Supplementary material for: A Rare Case Report of Nosocomial Urosepsis due to Klebsiella pneumoniae in a Preterm Neonate Complicated by Acute Cystitis
Source: Case Rep Infect Dis. 2026 Sep 12;2026:3455145. doi: 10.1155/crdi/3455145 (PMC13570409; doi:10.1155/crdi/3455145)
Supplement: Supplementary file 1 [file CRDI-2026-3455145-s001.pdf]

CARE case report guidelines

CARE Checklist of information to include when writing a case report

(cc) BY-NC-ND

Topic	Item	Checklist item description	Reported on Line
**Title**	1	The diagnosis or intervention of primary focus followed by the words "case report"	2
**Key Words**	2	2 to 5 key words that identify diagnoses or interventions in this case report, including "case report"	44 and 45
**Abstract (no references)**	3a	Introduction: What is unique about this case and what does it add to the scientific literature?	35 to 37
	3b	Main symptoms and/or important clinical findings	33 to 34
	3c	The main diagnoses, therapeutic interventions, and outcomes	36
	3d	Conclusion—What is the main "take-away" lesson(s) from this case?	38 to 41
**Introduction**	4	One or two paragraphs summarizing why this case is unique (**may include** references)	49 to 63
**Patient Information**	5a	De-identified patient specific information	71 to 73
	5b	Primary concerns and symptoms of the patient	90 to 91
	5c	Medical, family, and psycho-social history including relevant genetic information	74 to 78
	5d	Relevant past interventions with outcomes	80 to 88
**Clinical Findings**	6	Describe significant physical examination (PE) and important clinical findings	90 to 91
**Timeline**	7	Historical and current information from this episode of care organized as a timeline	80 to 114
**Diagnostic Assessment**	8a	Diagnostic testing (such as PE, laboratory testing, imaging, surveys)	92 to 103, 108 to 109
	8b	Diagnostic challenges (such as access to testing, financial, or cultural)	94 to 96 and 99
	8c	Diagnosis (including other diagnoses considered)	103
	8d	Prognosis (such as staging in oncology) where applicable	110 to 112
**Therapeutic Intervention**	9a	Types of therapeutic intervention (such as pharmacologic, surgical, preventive, self-care)	106 to 107
	9b	Administration of therapeutic intervention (such as dosage, strength, duration)	106 to 107
	9c	Changes in therapeutic intervention (with rationale)	105 to 106
**Follow-up and Outcomes**	10a	Clinician and patient-assessed outcomes (if available)	110 to 112
	10b	Important follow-up diagnostic and other test results	109
	10c	Intervention adherence and tolerability (How was this assessed?)	111 to 115
	10d	Adverse and unanticipated events	113 to 114
**Discussion**	11a	A scientific discussion of the strengths AND limitations associated with this case report	100 to 103, 172
	11b	Discussion of the relevant medical literature **with references**	125 to 168
	11c	The scientific rationale for any conclusions (including assessment of possible causes)	130 to 163
	11d	The primary "take-away" lessons of this case report (without references) in a one paragraph conclusion	165 to 172
**Patient Perspective**	12	The patient should share their perspective in one to two paragraphs on the treatment(s) they received	no reported
**Informed Consent**	13	Did the patient give informed consent? Please provide if requested ... Parents consent	**Yes** √ **No** ☐
